# Physical, Cognitive, Emotional, and Social Health Outcomes of Parents in the First Six Months after Childhood Critical Illness: A Prospective Single Centre Study

**DOI:** 10.3390/children11080948

**Published:** 2024-08-06

**Authors:** Pei-Fen Poh, Jan Hau Lee, Rehena Sultana, Joseph C. Manning, Matthew C. Carey, Jos M. Latour

**Affiliations:** 1 School of Nursing and Midwifery, Faculty of Health, University of Plymouth, Plymouth PL4 8AA, UK; matthew.carey@plymouth.ac.uk (M.C.C.); jos.latour@plymouth.ac.uk (J.M.L.); 2 Children’s Intensive Care Unit, KK Women’s and Children’s Hospital, Singapore 229899, Singapore; gmsljh@nus.edu.sg; 3 Paediatrics Academic Clinical Programme, Duke-NUS Medical School, Singapore 169857, Singapore; 4 Duke-NUS Medical School, Singapore 169857, Singapore; rehena.sultana@duke-nus.edu.sg; 5 Nottingham Children’s Hospital, Nottingham University Hospitals NHS Trust, Queen’s Medical Centre, Nottingham NG7 2UH, UK; joseph.manning@leicester.ac.uk; 6 School of Healthcare, College of Life Sciences, University of Leicester, Leicester LE1 7RH, UK; 7 Department of Nursing, Zhongshan Hospital, Fudan University, Shanghai 200437, China; 8 Curtin School of Nursing, Curtin University, Perth, WA 6102, Australia

**Keywords:** post-intensive care syndrome, paediatric intensive care, parents, health outcomes, longitudinal follow-up

## Abstract

Childhood critical illness can have long-term effects on families, but the extent and trajectory of recovery for parents are unknown. Using prospective longitudinal design, we describe the health outcomes of parents and their trajectory six months after paediatric intensive care unit (PICU) discharge. Parents reported health outcomes at PICU discharge (baseline), and 1-, 3-, and 6-months post-discharge. We used the Pediatric Quality-of-Life Family Impact Module, Patient Health Questionnaire-4, and post-traumatic stress disorder (PTSD) Checklist for DSM-5. The group-based trajectory model was used to identify recovery patterns. We included 128 parents of children aged 1 month to 18 years, admitted to the PICU for ≥48 h. Three post-discharge composite health trajectory groups were classified: 54 mild (42%), 68 moderate (53%), and 6 severe (4%). Parents in the mild and moderate groups returned to baseline health within the first 3 months, but those in the severe group exhibited worse outcomes at 6-months. The mean (SD) PICU stay durations for mild, moderate, and severe groups were 9 (16), 7 (10), and 38 (61) days; days of mechanical ventilation were 4 (5), 4 (7), and 18 (25) days; and readmission rates were 12 (22%), 23 (34%), and 4 (66%), respectively. Identifying these trajectories enables novel, targeted interventions for at-risk parents, underscoring the significance of integrated PICU follow-up care.

## 1. Introduction

Mortality rates during Paediatric Intensive Care Unit (PICU) admissions remain at around 4%, of which with every 100,000 children admitted, 4000 died [1]. As mortality decreases, the incidence of new morbidities among survivors has increased [2,3]. A systematic review encompassing 33 observational studies found that up to 60% of parents experienced symptoms of anxiety and 50% reported symptoms of depression up to three months post-discharge, with 30% meeting the criteria for post-traumatic stress disorder [4]. Approximately 8% of PICU survivors in Singapore experience new health issues following discharge, although the long-term effects and progression of these conditions are still not fully understood [5]. The impact of PICU admission on parents can last for years after childhood critical illness [6,7]. Family stressors are frequently related to the severity of the child’s illness, the PICU environment, changes in parenting roles, and the outcomes for the child [8,9,10]. Parents facing these stressors, compounded by disruptions in sleep, must quickly adapt to resumed caregiving roles once their child is discharged, navigating the ongoing recovery from critical illness [8,11,12]. Poor caregiver health may lead to suboptimal care for the child survivor and poorer health outcomes for the caregivers themselves [13,14]

The post-intensive care syndrome (PICS-p) conceptual framework illustrates the interdependence of health outcomes between parents and their children, integrating the family’s PICU experiences with ongoing family development and diverse recovery trajectories that may extend over decades [15]. However, the application of the PICS-p framework in parents still requires validation. Understanding these recovery trajectories, identifying at-risk parents and the associated risk factors are crucial steps in developing targeted interventions that can potentially improve outcomes related to post-intensive care syndrome [15].

Current understanding of the long-term impact on the physical, cognitive, social, and emotional health outcomes of parents following PICU admission remains limited. Given the significant effects of PICU stays on parents and, crucially, their ability to manage daily life responsibilities—including duties to the household and other children—there is a need for more research into long-term outcomes and health trajectories of these parents after their child’s critical illness [12]. Therefore, the aim of this study is to explore and understand the impact of PICU admission on parents’ longitudinal health outcomes in the first six months after PICU discharge.

## 2. Materials and Methods

### 2.1. Objectives

The primary aim of this study was to examine the health outcome trajectories of parents of PICU survivors over the first six months following discharge. Additionally, we sought to examine and compare the physical, cognitive, emotional and social health outcomes at the time of PICU discharge, and at 1-, 3-, and 6-months post-discharge.

### 2.2. Study Design

This is a prospective longitudinal observational study and part of the SHACK study (Trial registration: NCT04637113). Details of the SHACK study protocol, including sample size calculations, are available in the published protocol [16]. This study focuses on the long-term health outcomes of parents in the first six months after their child’s discharge from the PICU. Reporting of this study adheres to the guidelines set forth in the Strengthening the Reporting of Observational Studies in Epidemiology statement (STROBE) statement [17].

### 2.3. Participants

**Figure 1:**
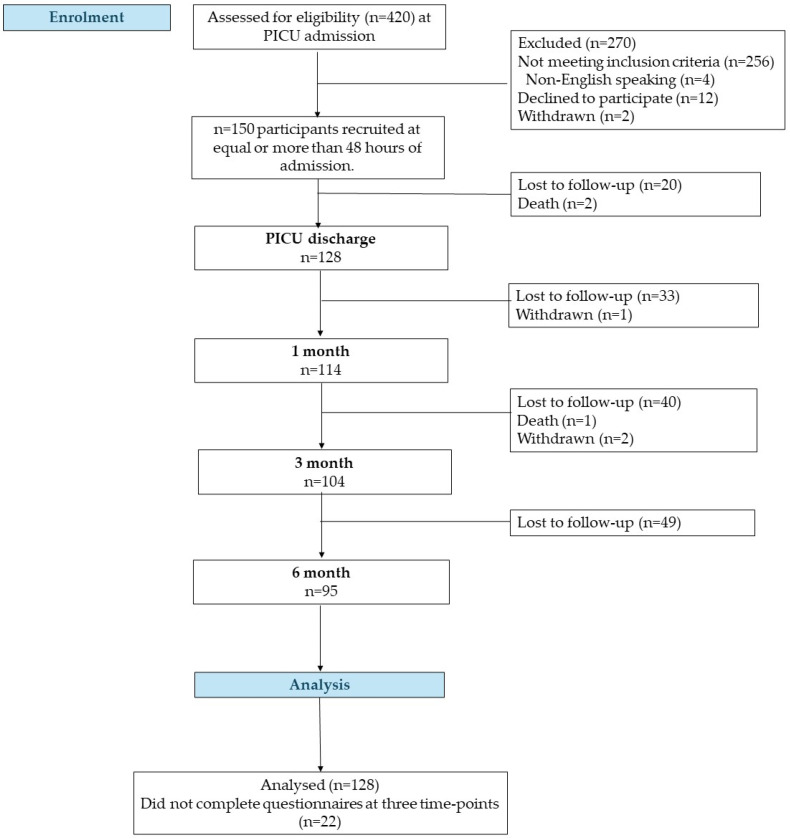
Participant recruitment flow diagram.

Eligible participants included parents of children aged 1 month to 18 years who were admitted to the PICU for a minimum of 48 h to ensure adequate exposure to the PICU environment and receive support for at least one organ. Parents of children who were under a do-not-resuscitate order at the time of admission were excluded from the study (Figure 1). To detect a 0.3 effect size difference in the Pediatric Quality of Life (PedsQL™) scores from PICU admission to six months post-discharge, a sample size of 110 was initially determined sufficient. However, a notable attrition rate of 40% (12/30) at the three-month follow-up prompted an ethical approval request to increase the recruitment target to 150 children, thereby ensuring adequate power for the six-month evaluations.

### 2.4. Measurement Instruments

Parents completed questionnaires at four time-points within the first six months following their child’s discharge from the PICU: at PICU discharge, 1-, 3-, and 6-months post-discharge. Further details regarding the psychometric properties of the instruments used can be accessed in the (Supplementary Material S1). The Pediatric Quality-of-life (PedsQL™) Family Impact Module is a 36-item instrument that assesses parental physical, cognitive, emotional, and social health [18]. Parents rated on a 5-point Likert scale ranging from 0 (never) to 4 (a lot), allowing for a total possible mean score between 0 and 100. Higher scores on this scale signify better health-related quality of life [18]. The Patient Health Questionnaire (PHQ)-4 and Diagnostic and Statistical Manual (DSM)-5 post-traumatic stress disorder (PTSD) checklist were used [19,20]. The PHQ-4 is a valid and reliable 4-item tool assessing anxiety and depression with a 4-point Likert scale, a total score of ≥3 for the first or last 2 questions suggests anxiety or depression respectively [19]. The DSM-5 PTSD Checklist consists of 20-items scored on a 5-point Likert scale from 0 to 4, with scores between 31 and 33 indicating probable PTSD [20]. The PedsQL™ Family Impact scale and the PHQ-4 were administered at each of the aforementioned time-points, whereas the DSM-5 PTSD checklist was assessed only at 3-and 6-months post-discharge. The Spiritual Coping Strategies, a 20-item instrument, contains the Religious and Spiritual coping strategies, items are rated on a 4-point Likert scale. Higher scores indicate more frequent use of these coping strategies measured once at PICU discharge [21].

### 2.5. Data Collection

Data collection encompassed baseline demographic characteristics and clinical data of the PICU survivors, which were extracted from the electronic medical records and entered into the JISC Online Survey platform (formerly known as Bristol Online Survey (BOS), 2023. To ensure comprehensive data collection, we issued weekly text via mobile reminders, up to a maximum of five times. Participants who did not respond after five reminders were considered to have missed that follow-up point but were permitted to continue participating in subsequent assessments. All data collected up to that point were included in the analyses.

### 2.6. Data Analysis

The group-based trajectory model (PROC TRACJ) was employed initially to determine the number of trajectory groups, thereby identifying specific trajectories for physical, cognitive, emotional, and social outcomes within the SHACK cohort [22]. The Bayesian Information Criterion (BIC) was subsequently utilised to ascertain the optimal number of groups. Iterative adjustments continued until the BIC indicated the most suitable model fit [23]. Latent groups were then consolidated, prioritizing the hierarchy of physical, cognitive, emotional, and social domain trajectories. These groups were categorised into mild, moderate, and severe trajectory classes and treated as a categorical variable.

Participant demographic data and health outcomes were summarised according to their trajectory group following their child’s PICU discharge. Continuous variables were presented as means with standard deviations (SDs), and categorical variables were shown as frequencies and percentages. Differences between continuous variables across the mild, moderate, and severe trajectory categories were analysed using analysis of variance (ANOVA), while categorical variables were assessed with the Chi-square test. Mixed model analyses were adjusted for predetermined variables, such as the child’s age and household income. The results of these analyses were reported as mean differences along with 95% confidence intervals (95% CI). No corrections for multiple comparison were accounted. All analyses were two-tailed, using SAS version 9.4, with *p*-values < 0.05 deemed significant.

### 2.7. Ethical Considerations

Parents provided written consent prior to research procedures. This study was approved by the SingHealth Centralised Institutional Review Board (IRB Ref: 2020/2997) on 18 November 2020 and the Faculty Research Ethics and Integrity Committee of the University of Plymouth (Ref: 2020-2506-1464) on 6 January 2021.

## 3. Results

### 3.1. Participants and Child’s Clinical Characteristics

In total, 128 parents completed at least three out of four follow-up measurements at 6 months after their children’s PICU discharge (Figure 1). The ethnic composition of the sample was reflective of Singapore’s demographic distribution, comprising 73 (57%) Chinese, 38 (29%) Malay and 8 (6%) Indian participants (2) [24]. Furthermore, the majority were mothers with mean age of 39 (SD = 8) years, held postgraduate qualifications, and the mean (SD) monthly household income ranged from S$5000–S$6800, falling below the national median of S$10,000 mean (SD), which is below the national median of S$10,000 (Ministry of Trade and Industry 2023) (Table 1) [25].

The PICU survivors had a mean age of 5.0 (SD = 5.4) years, and were primarily admitted for 58 (45%) medical, 29 (22%) cardiac and 14 (11%) surgical reasons. Mean Paediatric Index of Mortality (PIM) III score (severity of illness score) was 3 (SD = 4). Duration of PICU and hospital admission were 9 (SD = 22) and 32 (SD = 61) days, respectively (Table 2). We found no significant difference in parental outcomes based on whether the child had a pre-existing illness or was previously well. Of the parents, 88 (69%) completed all questionnaires at four time-points (compliant) while 39 (30%) failed to complete at least one time point (non-compliant). Children from non-compliant parents had significantly longer PICU (16 (SD = 38) vs. 6 (SD = 8), *p* = 0.015), hospital (54 (SD = 88) vs. 25 (SD = 41), *p* = 0.014) days and higher rates of hospital readmission (19 (48%) vs. 20 (22%), *p* = 0.004).

### 3.2. Overall Six-Month Health Outcomes Post-PICU Discharge

Health outcomes scores demonstrated improvement from PICU discharge, peaking at 3 months, with no significant improvement observed between the 3- and 6- months point (Supplementary Table S1). Significant improvement in the physical domain was noted only between PICU discharge and 1-month post-discharge −6.7 (95% CI −10.5, −3.01, *p* = 0.001), with no further improvement up to 6 months post-discharge (Supplementary Table S1). Within our cohort, an improvement of 4.5 points, meeting the Minimum Clinically Important Difference (MCID), was observed across all the PedsQL™ Family impact module domains within the first three months post-PICU discharge. Notably, 10/104 (9.6%) and 11/95 (11.6%) of parents were identified as meeting the criteria for PTSD diagnosis at 3- and 6-months, respectively. Throughout all time-points assessed, the proportions of parents meeting criteria for anxiety and depression were as follows: at PICU discharge anxiety 65 (51%) and depression 48 (38%); 1 month anxiety 19 (16%) and depression 13 (11%); 3 months anxiety 9 (8%) and depression 2 (1%); 6 months anxiety 11 (11%) and depression 5 (5%).

### 3.3. Trajectory-Based Categorization of Participants

**Figure 2:**
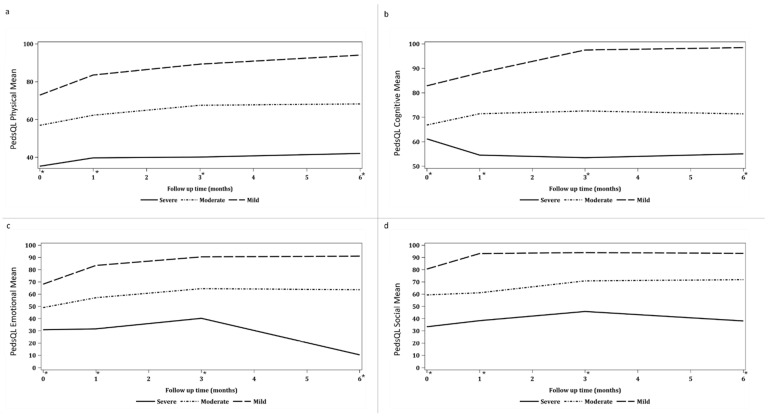
Trajectory of physical, cognitive, social, and emotional health, as indicated by *, at PICU admission, PICU discharge, and at 1, 3, and 6 months post-PICU discharge. (**a**) PedsQL Physical Mean, (**b**) PedsQL Cognitive Mean, (**c**) PedsQL Emotional Mean, (**d**) PedsQL Social Mean.

A total of 128 parents of PICU survivors were categorised based on their PICS-p domain scores, which focus on emotional and social health. Six months following their children’s PICU discharge these parents were categorised into three groups with priorities given to their emotional and social health domain scores: 54 (42%) as mild, 68 (53%) as moderate, and 6 (4%) as severe (Table 1). Parents in the mild and moderate categories had returned to their baseline level of health, whereas those in the severe category demonstrated significantly worse health outcomes compared to their baseline, as observed six months post-discharge (Figure 2).

### 3.4. Participant Characteristics across Health Outcome Trajectories

Given the small sample size (*n* = 6) of parents in the severe trajectory group, detailed characteristics are reported descriptively, and this subgroup was excluded from inferential analyses to prevent overinterpretation of underpowered statistical tests. The mean age of parents in the severe group was 34.0 (SD = 12.1) years, with 5 (83.3%) being married (Table 1). Their children were admitted to the PICU at a mean age of 4.0 (SD = 5.4) years with a mean PIM III score of 3.0 (SD = 4.2). The mean duration of PICU and hospital stay were 38 (SD = 61.5) and 105 (SD = 138.4) days, respectively (Table 2). In comparing the mild and moderate trajectory groups, 1.0 (1.9%) vs. 3.0 (4.4%) were reported as divorced (*p* = 0.030). Additionally, 19 (28%) parents in the moderate group reported higher incomes than 4 (7.5%) in the mild group (*p* = 0.042) (Table 1). However, no significant differences were observed in the child’s clinical characteristics between the mild and moderate groups.

### 3.5. Health Outcome Differences by Trajectory Group

**Figure 3:**
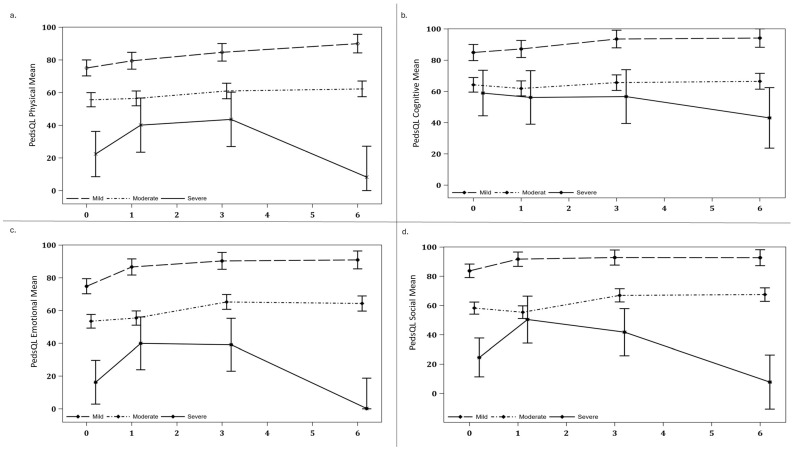
Mixed model analysis of differences in physical, cognitive, emotional, and social health, adjusted for child’s age and household income across PICU discharge, 1-, 3-, and 6-months after PICU discharge, across three groups (mild, moderate, severe). (**a**) PedsQL Physical Mean, (**b**) PedsQL Cognitive Mean, (**c**) PedsQL Emotional Mean, (**d**) PedsQL SocialMean.

As the classification of trajectories was based on the PedsQL™ Family Impact Module, focusing on emotional and social scores, inherent differences across these domains were anticipated observed between the mild and moderate trajectory groups across all PedsQL™ domains, including physical, cognitive, emotional, and social functioning, at each time point (*p* < 0.001) (Supplementary Tables S2 and S3). Nevertheless, no significant differences in parental spiritual coping behaviour were observed across the trajectory groups (*p* = 1.00). The moderate group consistently reported poorer health outcomes compared to the mild group throughout the study period. This trend aligns with the underlying premise of trajectory modelling, which aims to identify distinct subgroups based on their patterns of change over time (Figure 2 and Figure 3).

### 3.6. Longitudinal Changes in Health Outcomes by Trajectory Group

Utilizing mixed-model analyses, based on existing literature, adjusting for the child’s age and parents’ income, we examined the difference within the PICS-p domains across trajectory groups, finding both adjusted variables, such as child’s age and parents’ income, to be non-significant [26,27,28]. Notably, improvements in emotional (−11.79, 95% CI: −17.77, −5.81, *p* < 0.001) and social (−7.99, 95% CI: −13.95, −2.03, *p* = 0.009) domain scores were observed within the first month post-PICU discharge in the mild trajectory group, with no subsequent improvement (Table 3). Conversely, in the moderate group, improvements in the emotional (−9.82, 95% CI: −15.31, −4.34, *p* = 0.001) and social (−11.52, 95% CI:−16.98, −6.05, *p* < 0.001) domains were evident between 1- and 3-months post-discharge and plateaued at 3 months (Table 3 and Figure 2). For the severe trajectory group in the physical domain, mean scores decreased from 43.8 (SD = 12.5) at 3 months to 11.1 (SD = 19.2) at 6 months. For emotional domain, parents reported a mean score of 40.0 (SD = 20.0) at 3 months, which decreased to 3.3 (SD = 5.77) at 6 months. Similarly, social domain scores declined from a mean of 40.6 (SD = 19.7) at 3 months to 8.3 (SD = 14.4) at 6 months post-discharge (Figure 3).

## 4. Discussion

In this longitudinal study of parents of PICU survivors, we described the domains of the PICS-p conceptual framework, with emphasis on emotional and social health outcomes. Parents were categorised based on their health trajectories in the first six months after their child’s discharge from the PICU. Our study examined the association between parents’ health trajectories and a variety of determinates, including parental sociodemographic attributes and clinical characteristics of their children following PICU discharge. Additionally, we compared health outcomes across different time-points, employing latent class trajectory group modelling to delineate the patterns of parental recovery after a child’s critical illness.

Overall, physical health outcomes reached a plateau between PICU discharge and one month, aligning with our qualitative findings, which indicated that children predominantly required assistance with daily living activities within the first two weeks post-discharge [29]. By one month, most were able to resume school, suggesting that parental involvement may have reverted to pre-admission levels [29,30]. Previous studies have examined the physiological changes of parents, such as exhaustion, which may be attributable to factors like sleep deprivation within the hospital environment [4,31]. The plateau observed in cognitive, emotional, and social health by three months may be attributed to increased parental vigilance regarding their child’s health, concerns about full recovery, and apprehensions about the possibility of PICU readmission, culminating in a “return to normalcy” between 3- and 6-months post-discharge [29,32].

Our findings diverge from the existing literature, as most parents in our study returned to baseline within three months post-PICU, consistent with our qualitative findings [29]. Conversely, a scooping review including 71 studies reported enduring impacts on parents for up to 10 years post-PICU discharge [28]. For instance, a comprehensive systematic review of 33 studies highlighted a notable increase in mental health diagnoses among parents post-PICU [4]. This discrepancy may be attributed to our methodological approach of categorizing parents into distinct groups, revealing a smaller subset grappling with severe challenges.

While acknowledging the limitation of a small sample size in drawing robust conclusions, the descriptive statistics performed in this study offer valuable insights into this high-risk population [33]. The observed prolonged mechanical ventilation, extended PICU stays, and hospitalisation in our study may be attributed to the inherent uncertainty surrounding the child’s illness. This observation resonates with previous research indicating that such clinical factors significantly contribute to the stress and long-term impacts on caregivers [4,32,34]. Thus, despite the absence of inferential statistical support, these findings underscore a crucial area warranting clinical attention and further investigation, with the aim of mitigating long-term adverse outcomes within the severe trajectory group.

Identifying parents at risk of negative outcomes, including PTSD risk factors, during the PICU stay presents a critical opportunity for implementing targeted psychosocial interventions [35]. Our study reported a prevalence of approximately 10% for PTSD post-PICU, consistent with findings from a separate investigation examining PTSD rates in a cohort of 282 mothers post-ICU discharge [36]. Despite the seemingly modest percentage, research indicates a substantial increase in the likelihood of parents experiencing PTSD from pre-PICU to post-PICU, with a reported 87% rise in a cohort of 90,000 parents [36]. Moreover, evidence suggests a partner effect, wherein high maternal post-traumatic stress symptoms (PTSS) predict elevated PTSS in children [14]. Given these findings, proactive monitoring and intervention strategies may enhance parental health outcomes following PICU.

A comprehensive, family-centred care approach during PICU admission has the potential to mitigate the risk of post-intensive care syndrome in parents by fostering family visitation, active participation in care, presence during medical procedures, and guidance towards additional resources [37,38]. Strengthening family-centred care through increased engagement and collaboration has been shown to positively impact parental experiences [37,39]. Furthermore, the development of scalable, low-resource interventions for parents aimed at managing post-discharge stress could contribute to improved long-term recovery trajectories. Promising strategies may include cognitive behavioural therapy and mindfulness practices [35,40]. Incorporating minimally invasive screening for PICS among parents during their children’s PICU follow-up clinics could further enhance parental support and optimise long-term outcomes.

The strengths of this study lie in the innovative use of trajectory group modelling to analyse a cohort of parents across six months, alongside the utilization of the PICS-p and core outcome measures for data collection. This approach integrates the conceptual framework effectively and demonstrates the practicality of utilizing the PedsQL™ Family Impact Module for screening parental recovery processes. However, the study’s sample size presents a limitation, potentially influencing the robustness of statistical findings. Additionally, the imputation of missing data may have potentially skewed the results, particularly as the imputed cases tended to involve longer PICU stays, hospital admissions, and rates of readmission. Consequently, the severity of the PICS may have been underestimated. Most parents in our study had high education (28.1%) compared to the national average of 14%, which may limit the generalizability of our findings [24]. Acknowledging the underrepresentation of fathers, future studies could improve engagement by offering flexible participation options for both parents. While our study provides valuable insights into the long-term trajectories of parental health outcomes, the small sample size of the severe group (*n* = 6) limits our ability to draw robust conclusions or establish strong relationships. Future longitudinal studies with larger samples are essential to provide a more detailed examination of the factors linked to the various PICS-p trajectory classifications.

## 5. Conclusions

This study has identified three distinct recovery trajectories and the factors influencing these trajectories among parents of critically ill children within the first six months following PICU discharge. These findings underscore the importance of early identification and assessment of parents at risk during PICU admission. An important clinical implication of our findings is the need to implement screening and ongoing follow-up for these parents at PICU follow-up clinics to enhance their recovery and overall well-being. Given that parents in the severe trajectory group may not return to baseline within 6 months, future research should prioritise extended follow-up to better understand and support their long-term recovery and well-being.

## Figures and Tables

**Table 1:** Participant characteristics and baseline information categorised by trajectory groups.

Variables	Total (*n* = 128)	Mild Trajectory (*n* = 54)	Moderate Trajectory (*n* = 68)	Severe Trajectory (*n* = 6)
Parents’ age, mean (SD)	39.3 (8.1)	39.0 (9.5)	40.1 (6.4)	34.0 (12.1)
Mother, *n* (%)	91.0 (71.1)	33.0 (61.1)	53.0 (77.9)	5.0 (83.3)
Marital status, *n* (%)	128	54	68	6
Divorced/Separated	5.0 (3.9)	1.0 (1.9)	3.0 (4.4)	1.0 (16.7)
Married	118.0 (92.2)	48.0 (88.9)	65.0 (95.6)	5.0 (83.3)
Highest education, *n* (%)	128	54	68	6
Secondary	28 (22.0)	13 (24.0)	12 (19.0)	2 (33.0)
Polytechnic	33.0 (25.8)	16.0 (29.6)	15.0 (22.1)	2.0 (33.3)
Post-graduate	36.0 (28.1)	13.0 (24.1)	22.0 (32.4)	1.0 (16.7)
Undergraduate	31.0 (24.2)	12.0 (22.2)	18.0 (26.5)	1.0 (16.7)
Race, *n* (%)	128	54	68	6
Chinese	73.0 (57.0)	27.0 (50.0)	43.0 (63.2)	3.0 (50.0)
Indian	8.0 (6.3)	5.0 (9.3)	3.0 (4.4)	0
Malay	38.0 (29.7)	17.0 (31.5)	18.0 (26.5)	3.0 (50.0)
Others	9.0 (7.0)	5.0 (9.3)	4.0 (5.9)	0
Employment status, *n* (%)	128	54	68	6
Unemployed	91.0 (71.1)	40.0 (74.1)	49.0 (72.1)	2.0 (33.3)
Yes, full-time	14.0 (10.9)	5.0 (9.3)	8.0 (11.8)	1.0 (16.7)
Household income, SGD *n* (%)	128	54	68	6
<$1100	10.0 (7.8)	5.0 (9.3)	3.0 (4.4)	2.0 (33.3)
$1101–2250	17.0 (13.3)	10.0 (18.5)	6.0 (08.8)	1.0 (16.7)
$2251–4499	32.0 (25.0)	16.0 (29.6)	15.0 (22.1)	1.0 (16.7)
$5000–6800	46.0 (35.9)	19.0 (35.2)	25.0 (36.8)	2.0 (33.3)
$9000–11,249	11.0 (8.6)	3.0 (5.6)	8.0 (11.8)	0
>$11,250	12.0 (9.4)	1.0 (1.9)	11.0 (16.2)	0

Continuous variables were presented as mean (standard deviation (SD)). *p* values are calculated based on chi square test for categorical variables and Analysis of Variance (ANOVA) for continuous variables Mild: High baseline health scores, return to baseline. Moderate: Middle baseline scores, returning to baseline. Severe: Low initial health scores, further declining at 6 months.

**Table 2:** Patient demographics and clinical characteristics categorised by trajectory groups.

Variables	Total (*n* = 128)	Mild Trajectory (*n* = 54)	Moderate Trajectory (*n* = 68)	Severe Trajectory (*n* = 6)
Male, *n* (%)	71 (55.9)	30 (55.6)	38 (56.7)	3 (50.0)
Child’s age (years), mean (SD)	5 (5.4)	5 (5.1)	6 (5.6)	4 (5.4)
Weight (kg), mean (SD)	24 (20.0)	24 (21.0)	23 (18.8)	28 (26.4)
Height (cm), mean (SD)	111 (35.7)	111 (33.5)	111 (37.5)	108 (41.4)
Pre-existing illness, *n* (%)	56 (44.1)	23 (42.6)	30 (44.8)	3 (50.0)
PIM III illness severity score, mean (SD)	3 (4.01)	3 (4.46)	2 (3.62)	3 (4.2)
Admission diagnosis, *n* (%)				
Cardiac	29 (22.8)	14 (25.9)	15 (22.4)	0
Medical	58 (45.7)	26 (48.1)	30 (44.8)	2 (33.3)
Neurology	13 (10.2)	4 (07.4)	8 (11.9)	1 (16.7)
Oncology	13 (10.2)	4 (07.4)	8 (11.9)	1 (16.7)
Surgical	14 (11.0)	6 (11.1)	6 (9.0)	2 (33.3)
Use of inotropes, *n* (%)	52 (41.6)	26 (49.1)	25 (37.9)	1 (16.7)
Use of sedation, *n* (%)	79 (63.2)	32 (59.3)	43 (66.2)	4 (66.7)
Use of analgesia, *n* (%)	89 (70.1)	34 (63.0)	50 (74.6)	5 (83.3)
Mechanical ventilation (MV), *n* (%)	85 (66.9)	32 (59.3)	48 (71.6)	5 (83.3)
Duration of MV, days, mean (SD)	5 (8.91)	4 (5.07)	4 (7.99)	18 (25.8)
Tracheostomy creation, *n* (%)	5 (3.9)	1 (1.9)	2 (3.0)	2 (33.3)
ECMO, *n* (%)	4 (3.2)	1 (2.0)	3 (4.5)	0
Duration of ECMO, days, mean (SD)	6 (2.08)	4 (NA)	7 (1.53)	0
Early mobilisation, *n* (%)	67 (53.2)	29 (54.7)	35 (51.5)	3 (60.0)
Length of PICU admission, days, mean (SD)	9 (22.6)	9 (25.0)	7 (10.3)	38 (61.5)
PICU readmission, *n* (%)	17 (13.4)	9 (16.7)	7 (10.4)	1 (16.7)
Length of hospitalisation, days, mean (SD)	34 (61.09)	34 (69.1)	28 (36.7)	105 (138.4)
Hospital readmission, *n* (%)	39 (31.0)	12 (22.2)	23 (34.8)	4 (66.7)

*p* values are calculated based on chi square test for categorical variables and Analysis of Variance (ANOVA) for continuous variables. MV: mechanical ventilation, PICU: paediatric intensive care. Mild: High baseline health scores, return to baseline. Moderate: Middle baseline scores, returning to baseline. Severe: Low initial health scores, further declining at 6 months. SD: Standard deviation.

**Table 3:** Parental physical, cognitive, emotional, social outcomes over time by trajectory group adjusted for child’s age and monthly household income.

Group	Outcome	PICU Discharge vs. 1 Month Mean Diff (95% CI)	*p*-Value	1 Month vs. 3 Month Mean Diff (95% CI)	*p*-Value	3 Month vs. 6 Month Mean Diff (95% CI)	*p*-Value
Mild	Physical	−4.40 (−10.23, 1.43)	0.139	−5.15 (−11.33, 1.04)	0.103	−5.32 (−11.92, 1.27)	0.113
	Cognitive	2.77 (−15.41, 20.95)	0.765	−0.53 (−20.55, 19.49)	0.959	13.62 (−7.63, 34.87)	0.208
	Emotional	−11.79 (−17.77, −5.81)	<0.001	−3.73 (−10.08, 2.62)	0.249	−0.60 (−7.37, 6.17)	0.861
	Social	−7.99 (−13.95, −2.03)	0.009	−1.17 (−7.50, 5.16)	0.716	0.14 (−6.61, 6.88)	0.968
Moderate	Physical	−0.81 (−5.94, 4.32)	0.756	−4.53 (−9.87, 0.81)	0.096	−1.30 (−6.82, 4.22)	0.644
	Cognitive	3.82 (−1.04, 8.68)	0.123	−3.90 (−8.95, 1.15)	0.130	−0.81 (−6.03, 4.41)	0.761
	Emotional	−1.98 (−7.25, 3.29)	0.460	−9.82 (−15.31, −4.34)	0.001	0.98 (−4.70, 6.66)	0.734
	Social	2.80 (−2.45, 8.05)	0.294	−11.52 (−16.98, −6.05)	<0.0001	−0.52 (−6.18, 5.14)	0.857
Severe	Physical	−17.67 (−36.77, 1.43)	0.070	−3.54 (−24.57, 17.48)	0.740	35.38 (12.96, 57.81)	0.002
	Cognitive	−2.32 (−7.85, 3.21)	0.411	−6.34 (−12.19, −0.48)	0.034	−0.65 (−6.90, 5.60)	0.838
	Emotional	−23.70 (−43.21, −4.19)	0.017	0.84 (−20.61, 22.29)	0.939	38.95 (15.95, 61.95)	0.001
	Social	−25.85 (−45.28, −6.42)	0.009	8.59 (−12.77, 29.96)	0.429	34.07 (11.16, 56.98)	0.004

Results are expressed in terms of mean differences and 95% confidence interval (CI).

## Data Availability

The original contributions presented in the study are included in the article/Supplementary Material; further inquiries can be directed to the corresponding author.

## Supplementary Materials

The following supporting information can be downloaded at: https://www.mdpi.com/article/10.3390/children11080948/s1, Material S1: Data collection and measures; Table S1: Cohort physical, cognitive, emotional, social outcomes overtime; Table S2: Parents’ physical, cognitive, social and emotional health outcomes in mild, moderate, and severe trajectory groups; Table S3: Parents’ anxiety, depression and post-traumatic stress symptoms outcomes in mild, moderate, and severe trajectory groups.
