# Supplementary material for: Physical, Cognitive, Emotional, and Social Health Outcomes of Parents in the First Six Months after Childhood Critical Illness: A Prospective Single Centre Study
Source: Children (Basel). 2024 Aug 6;11(8):948. doi: 10.3390/children11080948 (PMC11353106; doi:10.3390/children11080948)
Supplement: Supplementary file 1 [file children-11-00948-s001.zip › children-3126696-supplementary.pdf]

Physical, cognitive, emotional, and social health outcomes of parents in the first 6-months after childhood critical illness: A prospective single centre study.

Supplementary materials

Electronic Supplementary Material S1. Data collection and measures.

The PedsQL™ Family Impact Module, a reliable tool (α = 0.9), assesses the impact of pediatric chronic health conditions on family functioning [1]. This instrument includes 36 items across 8 dimensions: physical, emotional, social, and cognitive functioning; communication; worry; daily activities; and family relationships. It uses a 5-point Likert scale ranging from 0 (never) to 4 (a lot), with possible scores from 0 to 100, where higher scores indicate better functioning.

The PTSD Checklist for DSM-5 (PCL-5) measures the 20 DSM-5 symptoms of post-traumatic stress disorder, with reliability ranging from α = 0.56 to 0.77 [2]. This 20-item self-report measure uses a 5-point Likert scale from 0 (not at all) to 4 (extremely), with symptom severity scores ranging from 0 to 80. A cut-off score between 31-33 suggests probable PTSD.

The Patient Health Questionnaire-4 (PHQ-4) is a 4-item questionnaire that assesses anxiety and depression. Scores range from 0 to 12 on a 4-point Likert scale from 0 (not at all) to 3 (nearly every day) [3]. A total score of ⩾ 3 on either the first or last 2 questions indicates anxiety or depression, respectively. Its reliability is acceptable at α = 0.75.

The Spiritual Coping Strategies (SCS) scale consists of 20 items divided into two subscales: religious coping strategies and spiritual coping strategies [4]. Respondents rate each item on a 4-point Likert scale from 0 (never used) to 3 (often used), with possible scores ranging from 0 to 60. Higher scores indicate greater use of spiritual and religious coping strategies, with reported reliabilities of α = 0.82 for religious coping and α = 0.74 for spiritual coping.

Electronic supplement Table S1. Cohort physical, cognitive, emotional, social outcomes overtime (N=128)

Outcome	PICU discharge vs 1 month	P-value	1 month vs 3 month	P-value	3 month vs 6 month	P-value
Physical	-6.78 (-10.56, -3.01)	0.001	-4.00 (-8.14, 0.13)	0.058	-2.81 (-7.21, 1.60)	0.209
Cognitive	-1.81 (-6.03, 2.40)	0.395	-4.41 (-7.97, -0.85)	0.015	-0.92 (-4.01, 2.16)	0.554
Emotional	-11.02 (-15.34, -6.69)	<0.001	-6.99 (-11.16, -2.82)	0.001	0.49 (-3.62, 4.60)	0.814
Social	-6.31 (-10.78, -1.84)	0.006	-6.49 (-10.63, -2.36)	0.002	0.00 (-3.80, 3.80)	1.000

Results are expressed in terms of mean differences and 95% confidence interval (CI).

Electronic supplement Table S2. Parent's physical, cognitive, social and emotional health outcomes in mild, moderate, and severe trajectory groups.

Variables	Total (N= 128) Mean (SD, min – max)	Severity status			Overall P-value
		Mild (n = 54) Mean (SD, min – max)	Moderate (n = 68) Mean (SD, min – max)	Severe (n = 6) Mean (SD, min – max)	
**PICU discharge**					
Parent PedsQL Physical	57.6 (20.93, 0 - 100)	67.9 (17.75, 33 - 100)	52.5 (18.55, 13 - 88)	23.6 (16.80, 0 - 46)	<.0001
Parent PedsQL Cognitive	71.0 (20.39, 0 - 100)	80.0 (18.94, 50 - 100)	64.9 (17.70, 20 - 100)	60.0 (32.09, 0 - 90)	<.0001
Parent PedsQL Emotional	55.5 (21.89, 0 - 100)	66.1 (16.54, 30 - 100)	49.8 (20.81, 0 - 100)	25.0 (25.69, 0 - 70)	<.0001
Parent PedsQL Social	62.5 (23.64, 0 - 100)	76.8 (19.43, 31 - 100)	54.7 (19.26, 13 - 100)	25.0 (22.01, 0 - 56)	<.0001
Spiritual coping scale religious	14.6 (7.86, 0 - 27)	14.4 (7.99, 0 - 27)	14.7 (7.90, 0 - 27)	16.5 (7.40, 6 - 27)	0.818
Spiritual coping scale non-religious	20.2 (7.41, 0 - 33)	20.1 (8.26, 0 - 33)	20.4 (6.70, 0 - 33)	17.8 (8.04, 9 - 33)	0.712
**1 month post PICU discharge**					
Parent PedsQL Physical	64.7 (20.47, 0 - 100)	78.5 (16.97, 38 - 100)	56.2 (15.68, 17 - 100)	37.5 (28.46, 0 - 67)	<.0001
Parent PedsQL Cognitive	72.9 (21.64, 0 - 100)	86.9 (15.90, 50 - 100)	63.3 (18.36, 20 - 100)	57.5 (38.41, 0 - 80)	<.0001
Parent PedsQL Emotional	67.1 (22.13, 0 - 100)	85.5 (15.22, 45 - 100)	55.2 (14.82, 0 - 90)	38.8 (31.72, 0 - 75)	<.0001
Parent PedsQL Social	69.4 (24.54, 6 - 100)	90.3 (13.59, 50 - 100)	55.2 (18.77, 6 - 94)	48.4 (29.92, 25 - 88)	<.0001
**3 months post PICU discharge**					
Parent PedsQL Physical	69.3 (22.25, 21 - 100)	83.4 (18.87, 29 - 100)	60.8 (19.10, 21 - 100)	43.8 (12.50, 25 - 50)	<.0001
Parent PedsQL Cognitive	77 (22, 25 - 100)	93 (15, 25 -100)	67 (20, 25 -100)	56 (13, 50 – 75)	<.0001
Parent PedsQL Emotional	73.9 (21.33, 10 - 100)	89.0 (15.23, 40 - 100)	65.3 (17.56, 25 - 100)	40.0 (20.00, 10 - 50)	<.0001
Parent PedsQL Social	75.7 (20.72, 13 - 100)	91.4 (12.87, 50 - 100)	66.7 (16.98, 25 - 100)	40.6 (18.75, 13 - 50)	<.0001
**6 months post PICU discharge**					
Parent PedsQL Physical	70.5 (23.12, 0 - 100)	87.7 (14.96, 50 - 100)	62.1 (17.24, 21 - 100)	11.1 (19.25, 0 - 33)	<.0001
Parent PedsQL Cognitive	77.2 (22.01, 25 - 100)	93.1 (14.2, 50 - 100)	68.1 (19.66, 25 - 100)	48.3 (22.55, 25 - 70)	<.0001
Parent PedsQL Emotional	72.1 (23.39, 0 - 100)	89.3 (14.96, 50 - 100)	64.2 (16.49, 30 - 100)	3.3 (5.77, 0 - 10)	<.0001
Parent PedsQL Social	74.7 (22.30, 0 - 100)	91.2 (13.54, 50 - 100)	67.2 (15.69, 31 - 100)	8.3 (14.43, 0 - 25)	<.0001

Continuous and categorical variables were presented as mean (standard deviation (SD), min - max) and frequency (percentages) respectively. $^{*}p<0.05$, $^{**}p<0.001$. Mild: High baseline health scores, returning to baseline at 6 months. Moderate: Middle baseline scores, also returning to baseline at 6 months. Severe: Low initial scores, further declining at 6 months. P values are based on analysis of variance (ANOVA) and Chi-square test for continuous and categorical variables respectively.

Electronic supplement Table S3. Parent's anxiety, depression and post-traumatic stress symptoms outcomes in mild, moderate, and severe trajectory groups.

Variables	Total (N= 128) Mean (SD, min – max)	Severity status			Overall P-value
		Mild (n = 54) Mean (SD, min – max)	Moderate (n = 68) Mean (SD, min – max)	Severe (n = 6) Mean (SD, min – max)	
**PICU discharge**					
Patient Health Questionnaire total	5.8 (3.80, 0 - 12)	4.6 (3.33, 0 - 12)	6.4 (3.80, 0 - 12)	9.3 (4.32, 2 - 12)	0.001
PHQ anxiety, n (%)	65 (51)	20 (37)	40 (59)	5 (83)	0.016
PHQ depression, n (%)	48 (38)	13 (24)	30 (44)	5 (83)	0.005
**1 month post PICU discharge**					
Patient Health Questionnaire total	2.6 (2.69, 0 - 12)	1.0 (1.47, 0 - 4)	3.5 (2.71, 0 - 12)	5.8 (4.19, 3 - 12)	<.0001
PHQ anxiety, n (%)	19 (16)	2 (4)	15 (23)	2 (50)	0.004
PHQ depression, n (%)	13 (11)	1 (2)	11 (17)	1 (25)	0.029
**3 month post PICU discharge**					
Patient Health Questionnaire total	1.4 (1.80, 0 - 8)	0.4 (0.88, 0 - 4)	2.0 (1.95, 0 - 8)	3.0 (2.00, 0 - 4)	<.0001
PHQ anxiety, n (%)	9 (8)	2 (4)	7 (12)	0 (0)	0.360
PHQ depression, n (%)	2 (1)	0 (0)	2 (3)	0 (0)	0.445
PTSD Checklist for DSM-5	10.3 (12.13)	3.9 (7.41)	13.8 (12.11)	26.5 (18.86)	<.0001
Met PTSD criteria, n (%)	10 (9.6)	1 (2)	7 (12)	2 (50)	0.005
**6 month post PICU discharge**					
Patient Health Questionnaire total	1.5 (2.45, 0 - 12)	0.1 (0.42, 0 - 2)	2.1 (2.51, 0 - 9)	7.0 (4.36, 4 - 12)	<.0001
PHQ anxiety, n (%)	11 (11)	0 (0)	9 (16)	2 (66)	<0.001
PHQ depression, n (%)	5 (5)	0 (0)	4 (7)	1 (33)	0.026
PTSD Checklist for DSM-5	11.6 (14.42, 0 - 61)	4.2 (7.65, 0 - 28)	15.3 (14.54, 0 - 61)	33.7 (30.01, 1 - 60)	<.0001
Met PTSD criteria, n (%)	11 (1)	0 (0)	9 (16)	2 (66)	0.001

Continuous and categorical variables were presented as mean (standard deviation (SD), min - max) and frequency (percentages) respectively. *p<0.05, **p<0.001. Mild: High baseline health scores, returning to baseline at 6 months. Moderate: Middle baseline scores, also returning to baseline at 6 months. Severe: Low initial scores, further declining at 6 months. P values are based on analysis of variance (ANOVA) and Chi-square test for continuous and categorical variables respectively.
